# Supplementary material for: Validation and psychometric properties of the Brazilian-Portuguese dispositional flow scale 2 (DFS-BR)
Source: PLoS One. 2021 Jul 13;16(7):e0253044. doi: 10.1371/journal.pone.0253044 (PMC8277065; doi:10.1371/journal.pone.0253044)
Supplement: S1 Appendix — Portuguese-Brazilian Long Version of the Dispositional Flow Scale 2. (PDF) [file pone.0253044.s001.pdf]

S1 Appendix. DFS-BR.

Por favor, responda às seguintes questões com relação à sua experiência na atividade escolhida. Estas questões estão relacionadas com os pensamentos e sensações que você pode experienciar durante a participação numa atividade. Você pode experienciar estas características em alguns momentos, a todo momento ou em nenhum momento. Não há respostas corretas ou erradas. Pense sobre quão frequentemente você experiencia cada característica durante sua atividade e então marque o número que melhor representa sua experiência.

Quando participa em: ____

Item1: Sinto-me desafiado, mas acredito que minhas habilidades irão me permitir enfrentar o desafio

◉ Nunca ○ Raramente ○ As vezes ○ Frequentemente ○ Sempre

Item2: Faço coisas corretamente sem pensar sobre como fazer

◉ Nunca ○ Raramente ○ As vezes ○ Frequentemente ○ Sempre

Item3: Sei claramente o que quero fazer

◉ Nunca ○ Raramente ○ As vezes ○ Frequentemente ○ Sempre

Item4: É muito claro para mim como estou me saindo na atividade

◉ Nunca ○ Raramente ○ As vezes ○ Frequentemente ○ Sempre

Item5: Minha atenção está focada inteiramente no que estou fazendo

◉ Nunca ○ Raramente ○ As vezes ○ Frequentemente ○ Sempre

Item6: Tenho um senso de controle sobre o que estou fazendo

◉ Nunca ○ Raramente ○ As vezes ○ Frequentemente ○ Sempre

Item7: Não estou preocupado com o que os outros podem estar pensando de mim

◉ Nunca ○ Raramente ○ As vezes ○ Frequentemente ○ Sempre

Item8: O tempo parece alterado (mais devagar ou mais rápido)

◉ Nunca ○ Raramente ○ As vezes ○ Frequentemente ○ Sempre

Item9: Realmente gosto da experiência que estou tendo

◉ Nunca ○ Raramente ○ As vezes ○ Frequentemente ○ Sempre

Item10: Minhas habilidades combinam com o desafio que estou experimentando

◉ Nunca ○ Raramente ○ As vezes ○ Frequentemente ○ Sempre

Item11: As coisas parecem acontecer automaticamente

◉ Nunca ○ Raramente ○ As vezes ○ Frequentemente ○ Sempre

Item12: Tenho um forte noção do que quero fazer

◉ Nunca ○ Raramente ○ As vezes ○ Frequentemente ○ Sempre

Item13: Estou ciente do quão bem estou fazendo

◉ Nunca ○ Raramente ○ As vezes ○ Frequentemente ○ Sempre

Item14: Não há esforço em manter a minha mente no que está acontecendo

Nunca Raramente As vezes Frequentemente Sempre

Item15: Sinto que posso controlar o que estou fazendo

Nunca Raramente As vezes Frequentemente Sempre

Item16: Não estou preocupado em como os outros podem me avaliar

Nunca Raramente As vezes Frequentemente Sempre

Item17: A forma como o tempo passa parece ser diferente da normal

Nunca Raramente As vezes Frequentemente Sempre

Item18: Amo a sensação relacionada ao que estou fazendo e quero sentir novamente

Nunca Raramente As vezes Frequentemente Sempre

Item19: Sinto que sou competente o suficiente para atender às demandas da situação

Nunca Raramente As vezes Frequentemente Sempre

Item20: Realizo a atividade automaticamente sem pensar muito

Nunca Raramente As vezes Frequentemente Sempre

Item21: Sei o que quero alcançar

Nunca Raramente As vezes Frequentemente Sempre

Item22: Tenho uma boa ideia do quão bem estou me saindo enquanto estou envolvido na tarefa/atividade

Nunca Raramente As vezes Frequentemente Sempre

Item23: Tenho total concentração

Nunca Raramente As vezes Frequentemente Sempre

Item24: Tenho um sentimento de total controle sobre o que estou fazendo

Nunca Raramente As vezes Frequentemente Sempre

Item25: Não estou preocupado com a forma como estou me apresentando

Nunca Raramente As vezes Frequentemente Sempre

Item26: Parece que o tempo passa rapidamente

Nunca Raramente As vezes Frequentemente Sempre

Item27: A experiência me deixa me sentindo ótimo

Nunca Raramente As vezes Frequentemente Sempre

Item28: O desafio e minhas habilidades estão em um nível igualmente alto

Nunca Raramente As vezes Frequentemente Sempre

Item29: Faço as coisas de forma espontânea e automática sem ter que pensar

◉ Nunca ○ Raramente ○ As vezes ○ Frequentemente ○ Sempre

Item30: Meus objetivos estão claramente definidos

◉ Nunca ○ Raramente ○ As vezes ○ Frequentemente ○ Sempre

Item31: Pela forma como as coisas estão progredindo percebo se estou indo bem

◉ Nunca ○ Raramente ○ As vezes ○ Frequentemente ○ Sempre

Item32: Estou completamente focado na tarefa em questão

◉ Nunca ○ Raramente ○ As vezes ○ Frequentemente ○ Sempre

Item33: Sinto-me em total controle das minhas ações

◉ Nunca ○ Raramente ○ As vezes ○ Frequentemente ○ Sempre

Item34: Não estou preocupado com o que os outros podem estar pensando de mim

◉ Nunca ○ Raramente ○ As vezes ○ Frequentemente ○ Sempre

Item35: Perdi minha noção de consciência sobre o tempo

◉ Nunca ○ Raramente ○ As vezes ○ Frequentemente ○ Sempre

Item36: A experiência é extremamente recompensadora

◉ Nunca ○ Raramente ○ As vezes ○ Frequentemente ○ Sempre
