# Supplementary material for: Validation and psychometric properties of the Brazilian-Portuguese dispositional flow scale 2 (DFS-BR)
Source: PLoS One. 2021 Jul 13;16(7):e0253044. doi: 10.1371/journal.pone.0253044 (PMC8277065; doi:10.1371/journal.pone.0253044)
Supplement: S2 Appendix — Portuguese-Brazilian Short Version of the Dispositional Flow Scale 2. (PDF) [file pone.0253044.s002.pdf]

S2 Appendix. DFS-Short BR.

Por favor, responda às seguintes questões com relação à sua experiência na atividade escolhida. Estas questões estão relacionadas com os pensamentos e sensações que você pode experienciar durante a participação numa atividade. Você pode experienciar estas características em alguns momentos, a todo momento ou em nenhum momento. Não há respostas corretas ou erradas. Pense sobre quão frequentemente você experiencia cada característica durante sua atividade e então marque o número que melhor representa sua experiência.

Quando participa em: ______________________________

Evento/Atividade

Item10: Minhas habilidades combinam com o desafio que estou experimentando

Nunca   Raramente   As vezes   Frequentemente   Sempre

Item20: Realizo a atividade automaticamente sem pensar muito

Nunca   Raramente   As vezes   Frequentemente   Sempre

Item21: Sei o que quero alcançar

Nunca   Raramente   As vezes   Frequentemente   Sempre

Item4: É muito claro para mim como estou me saindo na atividade

Nunca   Raramente   As vezes   Frequentemente   Sempre

Item32: Estou completamente focado na tarefa em questão

Nunca   Raramente   As vezes   Frequentemente   Sempre

Item24: Tenho um sentimento de total controle sobre o que estou fazendo

Nunca   Raramente   As vezes   Frequentemente   Sempre

Item7: Não estou preocupado com o que os outros podem estar pensando de mim

Nunca   Raramente   As vezes   Frequentemente   Sempre

Item17: A forma como o tempo passa parece ser diferente da normal

Nunca   Raramente   As vezes   Frequentemente   Sempre

Item36: A experiência é extremamente recompensadora

Nunca   Raramente   As vezes   Frequentemente   Sempre
