# Supplementary material for: Validation and psychometric properties of the Brazilian-Portuguese dispositional flow scale 2 (DFS-BR)
Source: PLoS One. 2021 Jul 13;16(7):e0253044. doi: 10.1371/journal.pone.0253044 (PMC8277065; doi:10.1371/journal.pone.0253044)
Supplement: Attachment — Submitted filename: Response to Reviewers.pdf [file pone.0253044.s006.pdf]

Responding to Reviewer Comments

Dear reviewers and editors of PLOS ONE, we would like to thank for your insightful comments and suggestions; each one has been carefully analyzed and considered in the new version of the paper. We also indicate in this letter the texts that were changed or included in the manuscript to answer all comments. We hope that all these changes fulfill the requirements to make the manuscript for its publication.

Looking forward to hearing from you.

Comment 1 – Reviewer 3: *There are some issues that, I believe, could contribute to improve the paper: ... 1. In general terms, the validation process of a test never really ends, nor could it be said that there is any definitive evidence regarding it. In other words, different validity evidences are obtained along the time (utility, content, construct, appearance, consequences, criteria, ...). For these reasons I would suggest including some ideas and reflexions about it in the conclusions and what could be the next step in order to obtain other validity evidences. On the other hand, in the forthcoming researches, studies about the interpretation of the score will be welcome and the study of cutoff points in the scale. This important issue in the adaptation and construction of a measurement instrument should be comment.*

Answer*:* Dear reviewer, Thank you for the suggestions. We included your observations in the section "Conclusion and future work" (lines 536-550) as detailed as follow.

> Validation and psychometric properties of self-reported questionnaires depend on the samples, when it has been applied, and its use [63,64]. In this sense, the evidence presented in this article is not definitive. Along the time, we expect to obtain different validity evidence, in which we will present more information about the strengths, weaknesses, and characteristics of the DFS-BR, in its long and short versions.
>
> In further tests and validations of the DFS-BR, we will conduct studies with focus in the interpretation of the scale values for different groups or subjects. The interpretation of scale values depends also the context, and although we presented here validation for general activities, each response has a specific activity. These activities and others (that will be gathered in the future) will define the context in which we will analyze and interpret the gathered values for the dispositional flow scale. Establishing cut-off points to define what mean low or higher values of dispositional flow scale is another relevant future study that we expect to conduct in the future. This kind of study is also an

important issue in the adaptation and construction of psychometric instrument that also depends on the population and context in which it is applied.

(… references …)

63. Price LR. Psychometric methods: Theory into practice. Guilford Publications;2016.

64. Sireci SG. On the validity of useless tests. Assessment in Education: Principles,Policy & Practice. 2016;23(2):226–235.

<u>Comment 2 – Reviewer 3</u>*: There are some issues that, I believe, could contribute to improve the paper: ... 2. There were missing data? If yes, how they were treated (deleted, multiple imputation method,…).*

Answer*:* Dear reviewer, missing and incomplete responses were automatically eliminated by the web application used by the participants to answer the items of the DFS-BR. We included this information in the section “Data analysis procedure” (lines 149-150) as detailed as follow.

> Incomplete responses were automatically removed from the dataset by the web application in which the respondents answered the items of the DFS-BR.

Comment 1 – Reviewer 3: *There are some issues that, I believe, could contribute to improve the paper: ... 3. In the descriptive statistics the skewness and kurtosis should be reported in order to justify the estimation method used.*

Answer: Dear reviewer, we added the indexes of skewness and kurtosis, as well as the statistics from Shapiro-Wilk test in the Table 1 as shown as follows.

Table 1. Descriptive statistics, normality test, and MSA of the dataset.

	M	SD	Mdn	Skew	Kurtosis	Statistic	p.val	MSAi
i1	3.737	0.967	4	-0.635	0.252	0.8714	<0.001	0.970
i2	3.562	0.904	4	-0.508	0.448	0.8707	<0.001	0.961
i3	3.106	0.978	3	-0.165	-0.190	0.9017	<0.001	0.959
i4	3.637	0.931	4	-0.621	0.321	0.8711	<0.001	0.959
i5	3.470	0.931	4	-0.403	0.013	0.8872	<0.001	0.958
i6	3.079	1.083	3	-0.053	-0.663	0.9159	<0.001	0.965
i7	3.523	0.898	4	-0.549	0.426	0.8701	<0.001	0.955
i8	3.003	1.183	3	0.063	-0.822	0.9156	<0.001	0.894
i9	3.555	1.012	4	-0.422	-0.143	0.8914	<0.001	0.827
i10	3.752	0.987	4	-0.651	0.110	0.8723	<0.001	0.953
i11	3.485	0.936	4	-0.648	0.448	0.8674	<0.001	0.962
i12	2.883	0.934	3	-0.058	-0.359	0.8994	<0.001	0.892
i13	2.925	0.951	3	-0.025	-0.320	0.9030	<0.001	0.884
i14	3.941	0.931	4	-0.833	0.617	0.8450	<0.001	0.934
i15	3.537	0.894	4	-0.415	0.081	0.8798	<0.001	0.950
i16	3.339	0.993	3	-0.339	-0.198	0.8994	<0.001	0.931
i17	3.380	0.901	3	-0.242	-0.052	0.8888	<0.001	0.963
i18	2.786	1.160	3	0.270	-0.690	0.9102	<0.001	0.908
i19	3.501	1.035	3	-0.356	-0.206	0.8926	<0.001	0.935
i20	3.718	0.945	4	-0.550	0.217	0.8732	<0.001	0.945
i21	3.167	0.880	3	-0.331	0.189	0.8772	<0.001	0.947
i22	2.814	0.948	3	0.099	-0.236	0.9011	<0.001	0.872
i23	3.539	0.870	4	-0.594	0.459	0.8620	<0.001	0.967
i24	3.627	0.954	4	-0.436	-0.083	0.8859	<0.001	0.935
i25	3.699	0.869	4	-0.696	0.618	0.8522	<0.001	0.960
i26	3.567	0.921	4	-0.461	0.109	0.8815	<0.001	0.962
i27	3.514	0.914	4	-0.393	0.064	0.8841	<0.001	0.977
i28	3.072	1.204	3	-0.032	-0.904	0.9154	<0.001	0.895
i29	3.123	1.116	3	-0.079	-0.594	0.9139	<0.001	0.908
i30	3.921	0.970	4	-0.749	0.252	0.8525	<0.001	0.955
i31	3.567	0.923	4	-0.409	0.132	0.8817	<0.001	0.938
i32	3.664	0.941	4	-0.567	0.210	0.8761	<0.001	0.940
i33	3.746	0.869	4	-0.683	0.802	0.8527	<0.001	0.967
i34	3.229	1.226	3	-0.160	-0.899	0.9098	<0.001	0.876
i35	3.627	1.065	4	-0.552	-0.151	0.8846	<0.001	0.788
i36	3.862	0.971	4	-0.887	0.766	0.8463	<0.001	0.951

__Comment 4 – Reviewer 3__: *There are some issues that, I believe, could contribute to improve the paper: ... 4. If the authors would like report an index to justify the adequacy of the sample size, in CFA there is one specific global fit index for that purpose (Critical N). Given the statistical procedure follow, the lines 147 to 153 could be deleted.*

Answer*:* Dear reviewer, we decided to not use the Critical N as part of fit indexes because it was not recommended by *Hu, L. T., & Bentler, P. M. (1999). Cutoff criteria for fit indexes in covariance structure analysis: Conventional criteria versus new alternatives. Structural equation modeling: a multidisciplinary journal, 6(1), 1-55.* Critical N indicates the small sample size at which chi-square would not be significant, but we reported (in the lines 150 to 155) the Kaiser-Meyer-Olkin and Bartlett's test of spherity as measurement of adequacy to perform factor analysis. In other words, they are not fit indexes (as the Critical N), but the requirements to start the performing of CFA.

__Comment 5 – Reviewer 3__: *There are some issues that, I believe, could contribute to improve the paper: ... 5. As the authors do, the polychoric correlations should be used and, at least, a description of its adequacy should be commented. A good reference to do that is: Holgado-Tello, F.P., Carrasco, M.A., Barrio, Mª.V., y Chacón, S. (2009). Factor Analysis of the Big Five Questionnaire using polychoric correlations in children. Quality & quantity, the International Journal of Methodology, 43(1), 75-85. If it is possible, the Bonferroni correction for the tests of adequacy of polychoric correlations should be reported.*

Answer*:* Dear reviewer, we added a briefly explanation of why we decided to use the polychoric correlation in the section "Data analysis procedure" (lines 164-167) as described as follows.

> Polychoric correlation reduces the effect of attenuation that occur in the measured latent variable when it used a small number of item levels to estimate this value [32], and it is frequently applied for self-report psychometric instruments, such as personality trait [33].
>
> …. (references)
>
> 32. Bonett DG, Price RM. Inferential methods for the tetrachoric correlation coefficient. Journal of Educational and Behavioral Statistics. 2005;30(2):213–225.

> 33. Holgado-Tello FP, Carrasco-Ortiz MA, del Barrio-Gándara MV, Chacón-Moscoso S. Factor analysis of the Big Five Questionnaire using polychoric correlations in children. Quality & Quantity. 2009;43(1):75–85.

Answer: Dear reviewer, we also decided not to report the Bonferroni correlation as test of adequacy, because our decision to use polychoric correlation was based only in theoretical foundation (cited in the reference [33] and explained above).

<u>Comment 6 – Reviewer 3</u>*:* *There are some issues that, I believe, could contribute to improve the paper: ... 6. The procedure followed to shorten the scale presents problems that should not be avoided. In this method, only reliability information is taken into account, and it has as a negative consequence that, if the reliability and discriminations is optimized, the validity suffers a deterioration. That is, the reliability increases at the cost of validity. This issue should be mentioned.*

Answer*:* Dear reviewer, thanks for the important observation. In the new version of our paper, this validity threat, and the way in which we attempted to deal with it was added to the section “Construct validity of the DFS-Short BR” (lines 348-355) as indicated as follows.

> Notice that the method uses to define the alternative shorten version of the DFS-BR (with the model *unidim2*) increases the reliability of the instrument, but it may cause deterioration in the validity of the scale. To avoid a high degradation, improving simultaneously the reliability and the fit indexes, we intended to minimize the number of items that were changed from the model *unidim1*. We only changed the original item in the short version when the difference between the highest loading for each factor and the loading of the original item was greater than 0.20 in the multicorrelated model.

<u>Comment 7 – Reviewer 3</u>*:* *There are some issues that, I believe, could contribute to improve the paper: ... 7. The adequacy of the model should be based on the global fit indexes. In this sense, if there are individual parameters that has not statistical relevance, but however, the global fit indexes are adequate, and they are conceptual congruence, there are not arguments to delete they from the model. Sometimes, the redaction in this sense is very restrictive. I could recommend taken into account, also, the sense of the items, and the global fit indices. In CFA perspective,*

the fit indexes should be related to the global of the model. In this sense, if the model fit, although there are individual parameters close to 0, or with low values, they could be considered relevant if they have coherence and congruence with the theoretical assumptions.

Answer*:* Dear reviewer, to avoid been restrictive, we rewrote some parts of the texts as detailed as follows.

- For the lines 256-259, we removed the details about the loadings of the item i35, and we justified its presence based on theoretical foundations.

> Feedback (UF); Clear Goals (CG); and Merging of Action and Awareness (MAA). All the items with the exception of the item i35 have ~~adequate factor loadings based on~~ loadings greater than 0.30 which is considered adequate according the criteria defined by Hair et al. (2013) [54]~~which state that~~, for sample size of more than $n > 350$~~, the factor loadings should have at least 0.30 for a statistically significant ($p < 0.01$). We decided to maintain the item~~ . Item i35 ~~in the factorial structure and for the rest of analysis because its estimated loading with 0.256 and Std.Error of 0.046 can be considered close to the cutoff value, being its 90% CI 0.180; 0.332~~is not removed to maintain congruence with the original version of the DFS-2 and the theoretical underlie of the flow theory.

- In the lines 385-388, we rewrote our previous observation making it to be like more suggestion that a need.

> Items with lowest discrimination values (i35, i12 and i29) also ~~had~~ show low item loadings in the nine multi-correlated model (see Fig. 1) indicating that they ~~are not valuable and should be modified for the next version~~ may be semantically improved in future versions of the DFS-BR to ~~improve its accuracy and~~ increase their discrimination power and improve their reliability.

- The lines 403-406 has been rewrote to make it more like a suggestion that a requirement.

> the extremes of the 5-point Likert scale for these items. One suggestion for the future versions of the DFS-BR is that these items ~~should be improved semantically to avoid~~ may be part of studies that improve their meanings, avoiding the discretion of

- In the lines 261-268, we removed the translations of each items to make the paragraph more readable that specific and tedious.

one item has close or greater values than 0.70 in the item loadings. ~~For the CSB, this item is~~ In the development of the original version of the DFS-2 [2], they were used to define the short version of the DFS-2. In our study, the items with these high values are the item i10 ~~(Item 10: *Minhas habilidades combinam com o desafio que estou experimentando* – My abilities match the challenge of what I am doing)~~for the CSB. For the MAA, these items are i11 ~~(Item 11: *As coisas parecem acontecer automaticamente* – Things just seem to happen automatically)~~ and i20~~(Item 20: *Realizo a atividade automaticamente sem pensar muito* – I do things automatically, without thinking too much)~~. For the CG, these items are i21 ~~(Item 21: *Sei o que quero alcançar* – I know what I want to achieve)~~ and i30~~(Item 30: *Meus objetivos estão claramente definidos* – My goals are clearly defined)~~. For the UF, this item is i4~~(Item 4: *É muito claro para mim como estou me saindo na atividade* – It is really clear to me how I am going)~~. For the CTH, all the four items i5, i14, i23 and i32 are close to 0.70. For the SC, the items with good loadings are i15 ~~(Item 15: *Sinto que posso controlar o que estou fazendo* – I feel like I can control what I am doing)~~ and i24~~(Item 24: *Tenho um sentimento de total controle sobre o que estou fazendo* – I have a feeling of total control over what I am doing)~~. For the LSC, the items with the best loadings are i7 ~~(Item 7: *Não estou preocupado* com o que os outros podem estar pensando de mim – I am not concerned with what others may be thinking of me),~~ and i16~~(Item 16: *Não estou preocupado em como os outros podem me avaliar* – I am not concerned with how others may be evaluating me)~~. For the TT, the item with factorial loadings of 0.70s was the i17~~(Item 17: *A forma como o tempo passa parece ser diferente da normal* – The way time passes seems to be different from normal)~~. For the AE, the item i27 ~~(Item 27: *A experiência me deixa me sentindo ótimo* – The experience leaves me feeling great)~~ and the item i36 ~~(Item 36: *A experiência é extremamente recompensadora* – The experience is extremely rewarding)~~ have good loadings.

Comment 8 – Reviewer 3: *There are some issues that, I believe, could contribute to improve the paper: ...8. GFI should be reported.*

Answer: Dear reviewer, thank you for your comment. We added the GIF (Goodness-of-Fit Index) and AGFI (Adjusted Goodness-of-Fit Index) to the Tables 2 and 6 (as shown below).

Table 2. Fit indexes of tested models in the validation of DFS-BR.

	χ^2	df	χ^2/df	CFI	TLI	GFI	AGFI	SRMR	RMSEA	RMSEA.CI
multicorrelated	2431.181	575	4.228	0.938	0.932	0.944	0.935	0.091	0.069	[0.066; 0.072]
2nd-order	2541.138	585	4.343	0.934	0.929	0.941	0.933	0.093	0.070	[0.067; 0.073]

Table 6. Fit indexes of tested models in the validation of DFS-BR.

	χ^2	df	χ^2/df	CFI	TLI	GFI	AGFI	SRMR	RMSEA	RMSEA.CI
unidim1	210.065	27	7.780	0.876	0.835	0.957	0.928	0.099	0.100	[0.088; 0.113]
unidim2	79.500	27	2.944	0.981	0.975	0.986	0.976	0.070	0.053	[0.040; 0.067]

A briefly explanation about these indexes and expected values was also added in the section “Data analysis procedure” (lines 178-181) as described as follows.

> (c) for the Goodness-of-Fit Index (GFI) and the Adjusted Goodness-of-Fit Index (AGFI), that takes into account the degrees of freedom of the model with respect to the number of variables, values close or greater than 0.90 were considered acceptable [34, 35];

Comment 9 – Reviewer 3: *There are some issues that, I believe, could contribute to improve the paper: ... 9. In general terms, the reliabilities of the scales are very low, this could be indicate that alternatives internal structures should be investigate.*

Answer*:* Dear reviewer, thank you for your observation. We added your comment in the section “Conclusions and future works” (lines 490 to 492) as indicated as follows.

> However, the reliabilities in many of these factors are low (values of 0.06) which may be indication of the presence of internal structures that should be investigated in future studies.
