# Supplementary material for: Validation and psychometric properties of the Brazilian-Portuguese dispositional flow scale 2 (DFS-BR)
Source: PLoS One. 2021 Jul 13;16(7):e0253044. doi: 10.1371/journal.pone.0253044 (PMC8277065; doi:10.1371/journal.pone.0253044)
Supplement: Attachment — Submitted filename: PONE-D-20-30707 review.docx [file pone.0253044.s004.docx]

**Reviewer Comments for Manuscript PONE-D-20-30707*:*** *Validation and psychometric properties of the Brazilian-Portuguese Dispositional Flow Scale 2 (DFS-BR)*

**General Remarks:**

I read this manuscript with great interest. Overall, the manuscript addresses an interesting and valuable issue. It also provides a measurement tool in the field of sports psychology. The study validated the psychometric properties of an adapted version of the DFS-2 for the Brazilian Portuguese language.

The study has a number of strengths. First, the information presented in this manuscript is consistent with the journal’s mission. The manuscript is clearly within the journal’s mission, and there is a relevance to the readership. Second, the study sample was chosen appropriately and described in sufficient detail for the results to be generalized. Finally, the data were analyzed with correct analysis methods and the findings obtained from the analysis are very strong.
